# Reply: “Comment on: Food for Bone: Evidence for a Role for Delta-Tocotrienol in the Physiological Control of Osteoblast Migration. *Int. J. Mol. Sci.* 2020, *21*, 4661”

**DOI:** 10.3390/ijms21186675

**Published:** 2020-09-12

**Authors:** Lavinia Casati, Francesca Pagani, Roberto Maggi, Francesco Ferrucci, Valeria Sibilia

**Affiliations:** 1 Department of Medical Biotechnology and Translational Medicine, Università degli Studi di Milano, 20129 Milano, Italy; lavinia.casati@unimi.it (L.C.); francesca.pagani@unimi.it (F.P.); 2 Department of Pharmaceutical Sciences, Università degli Studi di Milano, 20133 Milano, Italy; roberto.maggi@unimi.it; 3 Department of Health, Animal Science and Food Safety, Università degli Studi di Milano, 20133 Milano, Italy; francesco.ferrucci@unimi.it

Dear Editor,

We have carefully read the Letter to the Editor by Pang and Chin related to our paper entitled “Food for bone: evidence for a role for delta-tocotrienol in the physiological control of osteoblast migration” [1] published in the International Journal of Molecular Science.

We have some issues regarding the points raised by the authors.

1. The paper from Shen and colleagues 2018 [2] clearly shows the effect of dietary supplementation of tocotrienol in the suppression of bone resorption, probably mediated by the reduction of oxidative stress. Our statement “osteoporosis has been correlated with low intake, and serum levels of TTs” refers to this paper. We disagree with the authors that “*dietary tocotrienol level has not been shown to correlate with bone health, probably due to the absence of a reliable dietary questionnaire that could assess the tocotrienol intake*”, since Shen and colleagues (2018) have reported that a 12 week annatto-derived tocotrienol supplementation, previously used to examine the effects of tocotrienol on bone turnover, resulted in a significant increase in serum delta-tocotrienol levels in postmenopausal osteoporotic women [2].
2. We agree with the Authors that several PI3K inhibitors are available. However, LY294002 is widely used as inhibitor of PI3k (see 172 papers in Medline from 2010 to 2020). Its strong effect in inhibiting PI3kinase action is well established [3]. LY294002 was shown to decrease not only the level of Akt phosphorylation in rat osteoblasts but also its nuclear translocation [4]. We used the same LY294002 concentration previously used to study the effect of PI3K/Akt signaling on osteoblast differentiation and motility [5]. The authors indicate that the 10 uM LY294002 used in our experiments could induce cell death, thus interfering with cell migration. It is worth noting that the papers cited by the authors [6,7] refer to the proapoptotic action of LY294002 in cancer cells, an experimental condition not comparable to the physiological environment used in our study. Moreover, the paper from Tang and colleagues [8] showed that the LY294002 (10 uM), does not affect MC3T3E1 cell proliferation or apoptosis. Lastly, we have demonstrated that delta-tocotrienol phosphorylates Akt.
  Akt phosphorylation is involved in the regulation of several signaling pathways and transcriptional networks controlling osteoblast function [9]. In our study we aimed to examine the effects of δ-TT on MC3T3E1-cell migration, and we focused our attention on the Wnt/β-catenin pathway and Bone Morphogenetic Protein (BMP) signaling involved in osteoblast differentiation and the wound-induced cellular migration [10]. In particular, it has been reported that GSK3beta is a critical downstream substrate and effector of PI3K/Akt, and pAkt inhibits GSK3beta, promoting its phosphorylation [11]. In line with this assumption, LY294002 decreases both pAkt and β-catenin levels [12,13]. Even if the lack of measurements of the intranuclear translocation of β-catenin represents a limit of our study, as reported in our paper, we suggest that the canonical Wnt signaling pathway is involved in the effect of δ-TT on MC3T3-E1 cell migration for several reasons:
  - δ-TT treatment increased β-catenin transcriptional activity as detected by luciferase assay. Indeed, the plasmid coding for TCF-LEF-RE is activated only after the translocation and the binding of β-catenin.
  - δ-TT treatment increased the gene expression of β-catenin target genes such as BMP2 and OC.
  - Pretreatment with procaine, a local anesthetic drug, previously reported to inhibit the Wnt/β-catenin pathway [14,15], prevented the stimulatory action of δ-TT on cell migration. Our assumption is in line with previous, in vivo studies, showing that oral annatto TT supplementation reduced osteopenia caused by metabolic syndrome by reducing SOST and DKK1 levels, considered antagonist of the Wnt signaling pathway [16].
3. As reported by the authors, procaine in addition to an inhibitory action on the Wnt signaling pathway, exerts a DNA-methyltransferase inhibitory activity. This effect has been detected in colon cancer cells [17] not in physiological conditions. In fact, the inhibitory effects of procaine on both osteoblast differentiation [18] and on rat bone marrow mesenchymal stem cells are due to Wnt/β-catenin inactivation and independent on DNA methylation changes [15].
4. The authors wrongly reported that we have previously shown that δ-TT increases MC3T3E1-cell proliferation. In this paper, we have shown the effect of δ-TT on cell viability by using MTT assay [19], which is a biochemical assay used to evaluate the viability of the cells, not the proliferation. In more detail, MTT allows the assessment of the cell metabolic activity since the measure of the cell proliferation with MTT is related only to rapidly dividing cells, that is not the case of MC3T3-E1. So, the finding of a shift of G1/S in the cell cycle is not in contrast with our previous results.

As far as the use of NONIDET-P40 (is correct), it is widely considered a nonionic, nondenaturing detergent [20], used in several biochemical tests, including cell cycle analysis [21,22].

Considering the common interest with the authors in the studies regarding the effects of natural compound on bone cell activities, we hope for a future fruitful collaboration.

## References

[1] Casati L., Pagani F., Maggi R., Ferrucci F., Sibilia V. (2020). Food for Bone: Evidence for a Role for Delta-Tocotrienol in the Physiological Control of Osteoblast Migration. Int. J. Mol. Sci..

[2] Shen C.L., Yang S., Tomison M.D., Romero A.W., Felton C.K., Mo H. (2018). Tocotrienol supplementation suppressed bone resorption and oxidative stress in postmenopausal osteopenic women: A 12-week randomized double-blinded placebo-controlled trial. Osteoporos. Int. A J. Establ. Result Coop. Eur. Found. Osteoporos. Natl. Osteoporos. Found. USA.

[3] Vlahos C.J., Matter W.F., Hui K.Y., Brown R.F. (1994). A specific inhibitor of phosphatidylinositol 3-kinase, 2-(4-morpholinyl)-8-phenyl-4H-1-benzopyran-4-one (LY294002). J. Biol. Chem..

[4] Zhang Y., Zhang L., Yan M., Zheng X. (2007). Inhibition of phosphatidylinositol 3-kinase causes cell death in rat osteoblasts through inactivation of Akt. Biomed. Pharmacother..

[5] Zhang Z., Zhang X., Zhao D., Liu B., Wang B., Yu W., Li J., Yu X., Cao F., Zheng G. (2019). TGFbeta1 promotes the osteoinduction of human osteoblasts via the PI3K/AKT/mTOR/S6K1 signalling pathway. Mol. Med. Rep..

[6] Gong C., Liao H., Wang J., Lin Y., Qi J., Qin L., Tian L.Q., Guo F.J. (2012). LY294002 induces G0/G1 cell cycle arrest and apoptosis of cancer stem-like cells from human osteosarcoma via down-regulation of PI3K activity. Asian Pac. J. Cancer Prev..

[7] Jiang H., Fan D., Zhou G., Li X., Deng H. (2010). Phosphatidylinositol 3-kinase inhibitor(LY294002) induces apoptosis of human nasopharyngeal carcinoma in vitro and in vivo. J. Exp. Clin. Cancer Res..

[8] Tang S.Y., Xie H., Yuan L.Q., Luo X.H., Huang J., Cui R.R., Zhou H.D., Wu X.P., Liao E.Y. (2007). Apelin stimulates proliferation and suppresses apoptosis of mouse osteoblastic cell line MC3T3-E1 via JNK and PI3-K/Akt signaling pathways. Peptides.

[9] McGonnell I.M., Grigoriadis A.E., Lam E.W., Price J.S., Sunters A. (2012). A specific role for phosphoinositide 3-kinase and AKT in osteoblasts?. Front. Endocrinol..

[10] Gamell C., Osses N., Bartrons R., Ruckle T., Camps M., Rosa J.L., Ventura F. (2008). BMP2 induction of actin cytoskeleton reorganization and cell migration requires PI3-kinase and Cdc42 activity. J. Cell Sci..

[11] Koh S.H., Kim S.H., Kwon H., Kim J.G., Kim J.H., Yang K.H., Kim J., Kim S.U., Yu H.J., Do B.R. (2004). Phosphatidylinositol-3 kinase/Akt and GSK-3 mediated cytoprotective effect of epigallocatechin gallate on oxidative stress-injured neuronal-differentiated N18D3 cells. Neurotoxicology.

[12] Gupta A., Chatree S., Buo A.M., Moorer M.C., Stains J.P. (2019). Connexin43 enhances Wnt and PGE2-dependent activation of beta-catenin in osteoblasts. Pflug. Arch. Eur. J. Physiol..

[13] Wu X., Zheng S., Ye Y., Wu Y., Lin K., Su J. (2018). Enhanced osteogenic differentiation and bone regeneration of poly(lactic-co-glycolic acid) by graphene via activation of PI3K/Akt/GSK-3beta/beta-catenin signal circuit. Biomater. Sci..

[14] Gao Z., Xu Z., Hung M.S., Lin Y.C., Wang T., Gong M., Zhi X., Jablons D.M., You L. (2009). Procaine and procainamide inhibit the Wnt canonical pathway by promoter demethylation of WIF-1 in lung cancer cells. Oncol. Rep..

[15] Herencia C., Diaz-Tocados J.M., Jurado L., de Oca A.M., Rodriguez-Ortiz M.E., Martin-Alonso C., Martinez-Moreno J.M., Vergara N., Rodriguez M., Almaden Y. (2016). Procaine Inhibits Osteo/Odontogenesis through Wnt/beta-Catenin Inactivation. PLoS ONE.

[16] Wong S.K., Chin K.Y., Ima-Nirwana S. (2019). The Effects of Tocotrienol on Bone Peptides in a Rat Model of Osteoporosis Induced by Metabolic Syndrome: The Possible Communication between Bone Cells. Int. J. Environ. Res. Public Health.

[17] Villar-Garea A., Fraga M.F., Espada J., Esteller M. (2003). Procaine is a DNA-demethylating agent with growth-inhibitory effects in human cancer cells. Cancer Res..

[18] Marofi F., Vahedi G., Solali S., Alivand M., Salarinasab S., Zadi Heydarabad M., Farshdousti Hagh M. (2019). Gene expression of TWIST1 and ZBTB16 is regulated by methylation modifications during the osteoblastic differentiation of mesenchymal stem cells. J. Cell. Physiol..

[19] Casati L., Pagani F., Limonta P., Vanetti C., Stancari G., Sibilia V. (2019). Beneficial effects of delta-tocotrienol against oxidative stress in osteoblastic cells: Studies on the mechanisms of action. Eur. J. Nutr..

[20] Sinha S., Field J.J., Miller J.H. (2017). Use of substitute Nonidet P-40 nonionic detergents in intracellular tubulin polymerization assays for screening of microtubule targeting agents. Biochem. Cell Biol..

[21] Martinez P., Mallo D., Paulson T.G., Li X., Sanchez C.A., Reid B.J., Graham T.A., Kuhner M.K., Maley C.C. (2018). Evolution of Barrett’s esophagus through space and time at single-crypt and whole-biopsy levels. Nat. Commun..

[22] Rabinovitch P.S. (1994). DNA content histogram and cell-cycle analysis. Methods Cell Biol..

